# Management of Cerebrospinal Fluid Leakage after Microvascular Decompression Surgery: Clinical Strategy

**DOI:** 10.3390/life13081771

**Published:** 2023-08-18

**Authors:** Hyun-Seok Lee, Kyung-Rae Cho, Kwan Park, Chiman Jeon

**Affiliations:** 1 Department of Neurosurgery, Konkuk University Medical Center, Seoul 05030, Republic of Korea; 20220205@kuh.ac.kr (H.-S.L.); medicasterz@gmail.com (K.-R.C.); kwanpark@skku.edu (K.P.); 2 Department of Neurosurgery, Samsung Medical Center, Sungkyunkwan University School of Medicine, Seoul 06171, Republic of Korea; 3 Department of Neurosurgery, Korea University Ansan Hospital, Ansan 15355, Republic of Korea

**Keywords:** cerebrospinal fluid leakage, lumbar drainage, microvascular decompression, paradoxical rhinorrhea

## Abstract

(1) Background: Cerebrospinal fluid (CSF) leakage is one of the most common complications of microvascular decompression (MVD) surgery. Before fatal complications, such as intracranial infection, occur, early recognition and prompt treatment are essential. (2) Methods: The clinical data of 475 patients who underwent MVD surgery from September 2020 to March 2023 were retrospectively analyzed. In these patients, if there were any symptoms of CSF leakage, and if CSF leakage was evident, a lumbar drainage catheter was inserted immediately. (3) Results: CSF leakage was suspected in 18 (3.8%) patients. Five of these patients (1.1%) showed signs of CSF leakage during conservative management and subsequently underwent catheter insertion for lumbar drainage. The lumbar drain was removed after an average of 5.2 days, resulting in an average hospitalization of 14.8 days. In all 5 patients, CSF leakage was resolved without reoperation. (4) Conclusions: Our treatment strategy prevented the development of fatal complications. Close observation of the symptoms and postoperative temporal bone computed tomography and audiometry are considered to be good evaluation methods for all patients. If CSF leakage is certain, it is important to perform lumbar drainage immediately.

## 1. Introduction

Cerebrospinal fluid (CSF) leakage after microvascular decompression (MVD) via the posterior fossa is one of the most common and significant problems [1,2]. In neurosurgical practice, the rate of postoperative CSF leakage has been reported to range from 5% to 30% [1]. It is known that the incidence of CSF leakage after posterior fossa surgery is approximately six times higher than supratentorial surgery [3,4]. The incidence of CSF leakage after surgery using the retrosigmoid approach in patients with different pathologies, including hemifacial spasm, trigeminal neuralgia, tumors such as vestibular schwannomas, and cavernous malformations, has been reported to range from 0 to 22% [5,6].

To date, various techniques have been suggested to prevent and treat CSF leakage. Although the incidence of CSF leakage is lower than before, it is still a major complication of MVD surgery [7]. Incomplete closure of the dura during surgery may result in a pseudomeningocele. Furthermore, when the mastoid air cells open, abnormal channels are created, thereby resulting in CSF otorrhea or paradoxical CSF rhinorrhea [2]. Postoperative ear fullness and subjective decreased bone conduction are mostly due to intraoperative saline irrigation and are temporary. However, if symptoms persist, CSF leakage should be suspected. CSF leaks can lead to potentially fatal complications such as meningitis, encephalitis, and brain abscess [1]. These complications increase hospital costs and lengthen hospital stays [8,9]. Hospital costs for patients with postoperative CSF leakage are approximately 141% higher than those for patients without CSF leakage [3,4]. Therefore, the early and accurate diagnosis and treatment of CSF leakage is very important. CSF diversion via a lumbar drainage catheter is the first stage in the treatment of definite CSF leakage [10,11,12]. Although the incidence of CSF leakage has been drastically reduced in our center, it still occurs in a few patients. This article introduces our methods for diagnosing and treating CSF leakage.

## 2. Patients and Methods

A retrospective analysis was performed on the clinical data of all patients who underwent MVD surgery between September 2020 and March 2023. All surgeries were performed by a single surgeon (Kwan Park). This study was approved by the institutional review board. The study involved the clinical data of 475 MVD surgeries that were performed on patients with various neurovascular conflict syndromes. Among these surgeries, 427 were performed to treat hemifacial spasm. Additionally, 47 surgeries were performed to treat trigeminal neuralgia, and one surgery was performed to treat glossopharyngeal neuralgia. The study group consisted of 131 males and 344 females. The median age of the patients was 58 years, ranging from 19 to 82 years [Table 1].

**Figure 1:**
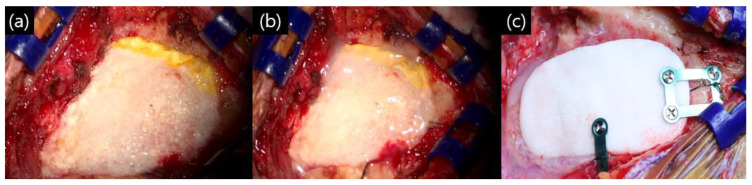
Our closing method after microvascular decompression (MVD): (**a**) after primary dural suturing with Duragen^®^, the dura was sealed with Tachosils^®^, a fibrin sealant; (**b**) fibrin glue was sprayed on the Tachosil^®^ layer; and (**c**) polymethyl methacrylate (PMMC) bone cement fixation with titanium plate and screw.

All patients were operated on in the park bench lateral position using the retro-mastoid suboccipital approach. The bone was drilled out. During the bone work process, as soon as the mastoid air cell opened, bone wax sealing was performed. After completion of the intracranial procedure and dural suturing, bone wax sealing was performed once again. The dural closing technique consisted of three layers. The collagen matrix dura substitute (Duragen^®^; Integra Lifescience, Princeton, NJ, USA) for dural sutures was based on the check valve theory: covering with an absorbable fibrin sealant patch (Tachosil^®^; Corza Health, Del Mar, CA, USA) and spraying fibrin glue (Tisseel^®^; Baxter, Deerfield, IL, USA). Finally, polymethyl methacrylate (PMMC) bone cement was fixed to the drilled out site (Figure 1). Immediately after surgery, brain computed tomography (CT) was performed to check the surgical site, and the patient was kept in the intensive care unit for observation on the day of surgery. A compression dressing was applied for 3 days after surgery, and temporal bone computed tomography (TBCT) was performed 3 days after surgery in all patients.

**Figure 2:**
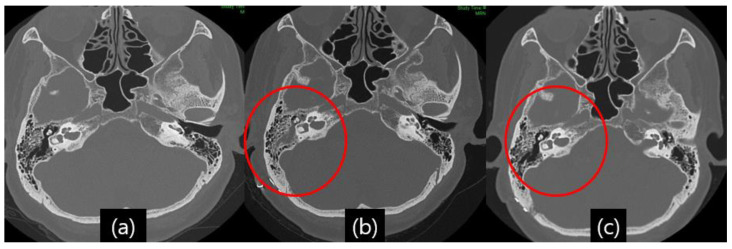
Illustrative case, 59-year-old female patient with right-side hemifacial spasm. Follow-up temporal bone computed tomography (TBCT): (**a**) preoperative TBCT, no abnormal findings; (**b**) follow-up TBCT 3 days after the operation, visible effusion inside the right mastoid air cell (red circle); (**c**) and after treatment of CSF leakage via lumbar drainage 45 days after the operation, the previously seen effusion inside the right mastoid air cell had disappeared (red circle).

Even with detailed closings, CSF leakage occurred in several patients, and the symptoms were clear otorrhea and rhinorrhea. Despite a suspected high false-positive rate [13], a glucose oxidase test was performed on patients with suspected CSF leakage. Since the glucose concentration in simple nasal secretions is usually approximately 10 mg/dL, it is easy to rule out whether CSF is admixed, and if the glucose concentration of the flowing fluid is more than 20 mg/dL, CSF leakage can be considered [14]. As mentioned, postoperative TBCT was performed in all patients who underwent MVD surgery to detect fluid effusion in the mastoid air cell, middle ear, and sphenoid sinus [Figure 2a,b].

In addition, otolaryngology consultation, including pure tone audiometry and speech audiometry, was performed in all patients. Among them, endoscopic ear examination was also performed when there was a large amount of middle ear effusion on TBCT. The possibility of CSF leakage was considered by combining the above results with the patients’ clinical symptoms, such as the time of occurrence and the amount of leakage, duration, and frequency.

After synthesizing the above results, if leakage was suspected or confirmed, a lumbar drainage catheter was inserted to divert the CSF, and the patient was placed on bed rest. When symptoms occur immediately after surgery, they are most likely due to saline residue in the mastoid aircells that were irrigated during surgery rather than CSF leakage. However, if postural rhinorrhea persists for more than three days, it is very likely to be due to CSF leakage and should be treated with a lumbar drain. Whereas when symptoms occur more than a few weeks after surgery, CSF diversion via lumbar drainage is less likely to result in dural healing. In these cases, revision surgery should be considered.

**Figure 3:**
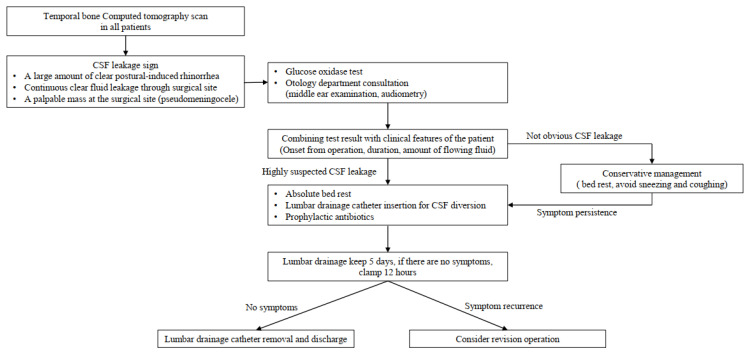
Our institution’s diagnosis and treatment policy for cerebrospinal fluid (CSF) leakage after microvascular decompression surgery.

Postural-induced rhinorrhea lasted for more than 3 days, and treatment was started with the possibility of CSF leakage in the mind. A lumbar drainage catheter was inserted into the third–fourth lumbar intervertebral space and maintained for 5 days with absolute bed rest. The daily CSF diversion amount was approximately 240 cc, 10 cc/h. In addition, intravenous first generation cephalosporin antibiotics for prophylaxis were used in combination with lumbar drainage. If symptoms of CSF overdrainage manifested, such as dizziness and headache, the drainage volume was reduced by 8 cc per hour, and if symptoms were still present, the drainage amount was reduced to 6 cc/h. We used a closed drainage system with a height gradient. The patients were moved to the sub-intensive care unit (sub-ICU) for careful observation of possible complications of a lumbar drain, such as overdrainage. In the sub-ICU, one nurse was assigned to each unit, allowing for more personalized patient care. In the absence of symptoms, the drainage volume was maintained. Five days after CSF drainage, the draining catheter was clamped from midnight to noon. After clamping for half a day, the presence of symptoms such as rhinorrhea and pseudomeningocele was checked. The catheter was removed if there were no symptoms. If there were symptoms, but fewer than there were before CSF diversion, we planned to maintain the catheter and CSF drainage for 2 more days, and if the symptoms were the same as before CSF drainage, we planned to consider revision surgery [Figure 3].

## 3. Results

In our study, which included a total of 475 patients, CSF leakage was suspected in 18 patients, representing a prevalence of 3.8%. Of the patients with suspected CSF leakage, 17 underwent MVD surgery to treat hemifacial spasm and 1 to treat trigeminal neuralgia. [Table 2]

**Figure 4:**
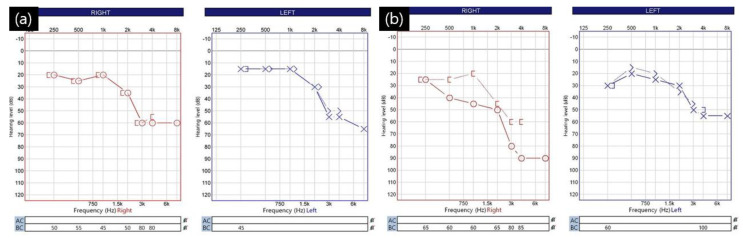
Audiometry of a 59−year−old female patient with right-side hemifacial spasm with cerebrospinal fluid leakage: (**a**) preoperative audiometry. Hearing difficulty in the high−pitched region but bilaterally symmetrical audiometry findings; (**b**) follow-up audiometry at 4 days after the operation shows obvious right-side conductive hearing loss of pure tone audiometry (PTA). The patient had no change in brain stem auditory potential (BAEP) during the operation, and there was no hearing loss of the sensorineural type seen during the vestibulocochlear nerve injury during surgery.

Of the 18 patients with suspected CSF leakage, 5 (1.1%) had a strong suspicion of CSF leakage, and all of these patients had lumbar drainage catheters; the remaining 13 patients did not have a lumbar drainage catheter. In all 18 patients, the mastoid air cell opened during surgery. Symptoms related to CSF leakage occurred within an average of 3.3 days (range: 2–6 days) after surgery. Among the 13 patients who did not have a lumbar drainage catheter, the average length of hospital stay was 8.2 days (range: 7–13 days). Meanwhile, among the five patients who underwent lumbar drainage, the average time from surgery to catheter insertion was 5.2 days (range: 3–8 days). The lumbar drainage catheter was maintained in the subarachnoid space for an average of 5.2 days (range: 4–6 days). The average length of stay for these five patients was 14.8 days (range: 13–19 days). All five patients who underwent lumbar drainage complained of hearing difficulties. Following consultation with an otolaryngologist, they were diagnosed with conductive hearing loss [Figure 4].

As a result, both patients with a lumbar drainage catheter and those without a catheter due to mild symptoms recovered without reoperation and without recurrence of CSF leakage symptoms and did not progress to meningitis and brain abscess (Table 3).

### Illustrative Case

**Figure 5:**
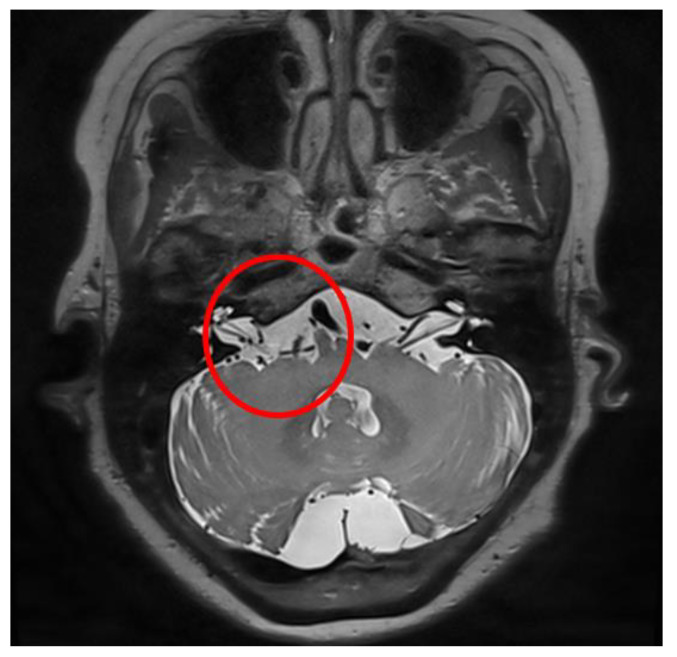
A 59-year-old woman with right HFS. Preoperative proton density-weighted (PD-weighted) magnetic resonance imaging (MRI). In the exit zone of the right facial nerve root, the right vertebral artery (VA) and anterior inferior cerebellar artery (AICA) were compressed in a tandem pattern (red circle).

A 59-year-old woman developed right eye and mouth twitching 2 years prior to admission and was diagnosed with HFS. After 3 botulinum toxin treatments, she was referred to our institution for MVD surgery. The offending vessel was a tandem type of right AICA and VA [Figure 5]. After decompression, the lateral spread response disappeared, the dura was sutured with a triple layer suturing technique, and there were no special intraoperative problems. The patient complained of postoperative headache and ear fullness. Postoperative follow-up TBCT showed effusion of the right mastoid air cells [Figure 2b], and the audiometry examination showed conductive hearing impairment [Figure 4]. First, the patient was placed on bed rest and observed for one day, but their symptoms did not improve, so a lumbar drainage catheter was inserted and drained at 8 cc per hour for 3 days. On the fourth day after placement of the CSF drain, the drain was clamped from midnight and observed until noon, and the drain was removed since there were no previous symptoms of CSF leakage. The patient was discharged 1 day later. Approximately 1 month after discharge, the patient visited the outpatient department, previous rhinorrhea and ear fullness had disappeared, and effusion of the mastoid air cells was no longer present on follow-up TBCT [Figure 2c]. The postoperative facial spasm had also disappeared.

## 4. Discussion

In the evaluation of CSF leakage, it is important to accurately assess the patient’s symptoms and initiate prompt evaluation and treatment when symptoms develop. We also suggested that pre- and postoperative TBCT and audiometry tests are helpful in evaluating CSF leakage. And diagnostic tests can be used in coordination with clinical symptoms to evaluate CSF leakage.

The first and simplest test to assess CSF leakage is a glucose oxidase test because it is easy to carry out and not expensive. However, it is not recommended as a confirmatory test due to its high false-positive and false-negative rates [13]. This is because glucose can be detected in normal nasal secretions or tears, in the respiratory tract of diabetics, in stress-induced hyperglycemic conditions, and in nasal epithelial inflammation due to the common cold [15]. Therefore, it should be considered as a reference that should be interpreted in conjunction with the patient’s clinical symptoms; thus, this test alone should not be used to influence the decision to perform an invasive procedure.

Other clinicopathologic diagnostic tests may include a beta-2 transferrin (β-2 trf) or a beta-trace protein (β-TP) test. Beta-2 transferrin is a specific isoform of transferrin that is found only in the CSF, vitreous humor of the eyeball, and perilymph. The presence of beta-2 transferrin in CSF can be considered a reliable indicator because it is not present in other body fluids and is not affected by the patient’s condition. The most commonly used detection technique is immunofixation electrophoresis. This test has been reported to have a sensitivity of 99% and a specificity of 97% [16], and other studies have reported a sensitivity of 84% and a specificity of 100%, with 100% positive and 95% negative predictive values [13]. Importantly, however, the beta-2 transferrin test is expensive, takes a long time to obtain results, and is not accessible in all institutes. Beta-trance protein (β-TP) is produced in the leptomeningeal epithelial cell linings and choroid plexus of the ventricle [16]. For the detection of beta-trace proteins, relatively small samples (200 μL) can be used to determine results in a short time (approximately 20 min). CSF leakage can be assessed by comparing the concentration between CSF and serum. This is because the concentration of β-TP in CSF is over 30 times greater than that in serum, known to be 0.46 ± 0.13 mg/L in serum and 14.6 ± 4.6 mg/L in CSF [13]. However, detection cut-off values have been reported to vary widely. Risch et al. reported that setting a cut-off value of 1.11 mg/L for beta-trace protein effectively identified patients with CSF leaks with good sensitivity (93%) and specificity (100%) [17]. Beta-2 transferrin and beta-trace protein are good diagnostic markers, but they have limitations and are not yet widely used. These two tests were not performed at our institution because they are time-consuming and costly, and we were not able to perform the tests ourselves. Nevertheless, in institutions where these tests are available, we expect that a diagnosis of CSF leakage can be based on a combination of clinical presentation and the results of these tests.

In our institute, there were 18 suspected CSF leakage cases (3.8%), and five patients (1.1%) showed a very high possibility. In several studies, the various etiologies of CSF leakage in surgery via the retro-mastoid suboccipital approach include vascular lesions, tumors, and neurovascular compression, and the incidence of cases with such etiologies is 0–22% [5,6,18]. Our surgery team had previously used the “plugging muscle” method to suture the dura mater [19]; however, due to the risk of causing damage to the muscle layer due to resection of the muscle and the risk of causing an intracranial infection due to the insertion of an intradural foreign body, we switched to the ‘triple layer closing technique’ mentioned above, which was also found to be quite effective in preventing CSF leakage. In addition to this method, the paradoxical case of rhinorrhea caused by CSF leakage from the surgical dural defect occurs through the mastoid air cells and Eustachian tube, and it is very important to seal the mastoid air cells with bone waxing.

CSF leakage may occur as surgical site leakage, including pseudomeningocele, ottorhea, or rhinorrhea [8,20,21]. CSF leakage through a surgical wound is diagnosed when clear fluid continuously leaks through a skin incision site when CSF collects palpably in the subcutaneous layer of the surgical site [20,21]. In our cases, there were no cases of such percutaneous CSF leakage or pseudomeningocele. If there were patients in our group with CSF leakage, the mastoid air cells were not opened during surgery, and it is thought that the symptoms appeared as a surgical site leakage or a pseudomeningocele rather than rhinorrhea or hearing difficulty. Therefore, it is thought that not only rhinorrhea and otorrhea but also the surgical wound should be continuously checked after surgery.

We analyzed the results of imaging tests such as TBCT, consulted with an otolaryngologist, and assessed the patients’ clinical features to determine the possibility of CSF leakage and proceed with treatment. Among these, it was relatively important to evaluate the effusion of mastoid air cells by performing a TBCT for all patients 3 days after MVD surgery. Normally, all patients who undergo cranial surgery undergo a brain CT immediately after surgery to evaluate the presence or absence of postoperative hemorrhage and the condition of the surgical site, but in addition, MVD patients undergo a TBCT on the third day after surgery. In patients with intraoperative mastoid air cell opening, the possibility of CSF leakage was assessed to be low even if the patients had symptoms such as slight rhinorrhea or hearing difficulty when there was no middle ear effusion or pseudomeningocele on follow-up TBCT. Accordingly, it is considered important to check temporal bone computed tomography (CT) scans for signs of possible subcutaneous CSF collection and the effusion of mastoid air cells. For that reason, we checked all patients by TBCT on the third day after surgery, which was useful for patient evaluation [Figure 2]. In addition, all patients underwent pre- and postoperative pure tone audiometry (PTA) and speech audiometry (SA) and consultation with an otolaryngologist. We performed brain stem evoke potential (BAEP) monitoring during all of our MVD surgeries, which helped us assess the development of sensorineural hearing loss due to intraoperative injury. We suggest that postoperative audiometry evaluation is necessary because it helps to recognize symptoms caused by the effusion of mastoid air cells and to predict the prognosis of surgery-related hearing difficulty [Figure 4].

The insertion of a lumbar drainage catheter was first mentioned by Voursh in 1960 [22]. A lumbar drainage catheter is inserted into the subarachnoid space of the lumbar spinal cord and diverts the flow of CSF outside of the patient. In the neurosurgery department, indications for lumbar drainage include the diagnosis and treatment of hydrocephalus and postoperative or post-traumatic CSF leakage, the prevention of CSF fistula formation, and the reduction of CSF volume to shrink the brain prior to cranial surgery [23]. Although there are many opinions about lumbar drainage catheter insertion in patients with CSF leakage, many studies have reported its advantage over conservative treatment [10,12,20]. According to the reports of several authors, 85–94% of CSF leakages were resolved when treated with lumbar drainage catheter insertion [22].

Lumbar drainage catheter insertion in itself is a fairly invasive procedure. In addition to mild complications such as headache, nausea, and vomiting, fatal complications such as meningitis, intracranial hemorrhage, and brain herniation due to the overdrainage of CSF may occur [23,24,25,26]. Therefore, it is important to ensure that the amount and rate of CSF drainage remain constant hourly to prevent the excessive drainage of CSF.

Invasive catheterization, such as lumbar drainage and extraventricular drainage, is closely related to the occurrence of central nervous system infections, such as meningitis.

Liang et al. analyzed risk factors for lumbar drain-related infections. Out of a total of 629 patients with a lumbar draining catheter, infection occurred in 36 (5.7%) patients, with significant risk factors being the in-dwelling of a lumbar drainage catheter for more than 4 days (*p* = 0.0037), longer hospitalization (≥21 days: *p* = 0.0007; 16–20 days: *p* = 0.0032), and CSF leakage through the lumbar puncture site (*p* < 0.0001). The predominant pathogen was methicillin-resistant coagulase-negative staphylococcus (MRCNS) [27]. Therefore, it is important to prevent CSF oozing from the puncture site after insertion of the lumbar drainage catheter. It is also important to diagnose CSF leakage as soon as possible and initiate treatment to keep the length of hospital stay as short as possible. In our study, there were no cases of infection when the lumbar drainage catheter was maintained for 4 to 6 days.

We also used first-generation cephalosporin prophylactic antibiotics during the period of lumbar drainage catheter placement to prevent infections associated with lumbar drain catheters. However, it is important to recognize that the use of prophylactic antibiotics remains controversial. Some authors suggest that prophylactic antibiotics are effective in reducing the incidence of meningitis; they claim that these antibiotics help to prevent the development of infection by eliminating or suppressing potential pathogens. On the other hand, there are authors who express concern about the use of prophylactic antibiotics. They argue that these antibiotics have the potential to disrupt normal microbial communities. This disruption can lead to an increased risk of infections, including meningitis. Critics of prophylactic antibiotic use emphasize the increased potential for the development of antibiotic-resistant bacterial infections, the increased risk of drug side effects, and more [9,28,29]. We and other authors note that a well-designed prospective study of antibiotic use is needed [27,30,31].

Limitations of this study include a small sample of patients with CSF leakage and the need for larger studies to identify more effective management strategies. Further analysis is needed to identify the risk factors for CSF leakage after MVD surgery. As more cases of CSF leakage accumulate, a comprehensive risk assessment for prognosis may be possible.

## 5. Conclusions

Efforts have been devoted to mitigating CSF leaks during cranial surgery; however, complete prevention of CSF leaks remains challenging, particularly in MVD via posterior fossa approach. Nonetheless, significant progress has been made in reducing the occurrence of CSF leaks, thereby leading to a reduced risk of fatal complications such as cerebral abscess and meningitis. It is crucial to emphasize the importance of early and accurate diagnosis, as well as the importance of effective CSF diversion through a lumbar draining catheter.

While our methods have proven to be successful in preventing serious complications associated with CSF leaks, methods to ensure complete prevention remain elusive. In our study, all patients with CSF leakage had conductive hearing loss, so it is helpful to follow up with temporal bone CT after surgery and perform pre- and postoperative audiometry on all patients for an early diagnosis. The lumbar drainage catheter was maintained for an average of 5.2 days, draining approximately 200 cc of CSF per day.

In summary, while complete prevention of CSF leaks during MVD surgery remains a challenge, significant progress has been made in reducing their occurrence and associated complications. Early diagnosis, CSF drainage with lumbar drains, and implementation of the triple-layer suturing technique for closing the dura have been proven to be beneficial; however, further research is needed to improve diagnostic approaches, determine optimal protocols, and identify risk factors for CSF leakage in larger patient populations.

## Figures and Tables

**Table 1:** Clinical characteristics of the patients.

**Total patients**	
Median age, years (range)	58 (19–82)
Male:Female	131:344
Left:Right	223:252
Suspected CSF leakage	18 (3.8%)
Lumbar drainage catheter insertion	5 (1.1%)
**Disease**	
Trigeminal neuralgia	47 (9.9%)
Hemifacial spasm	427 (89.9%)
* Glossopharyngeal neuralgia	1 (0.0%)
**Trigeminal neuralgia**	
Median age, years (range)	63.5 (28–80)
Male:Female	16:31
Left:Right	17:30
Suspected CSF leakage	1
Lumbar drainage catheter insertion	0
**Hemifacial spasm**	
Median age, years (range)	58 (19–82)
Male:Female	115:312
Left:Right	227:200
Suspected CSF leakage	17
Lumbar drainage catheter insertion	5

* The glossopharyngeal neuralgia patient was a 65-year-old female, left side, with no CSF leakage symptoms. CSF, cerebrospinal fluid.

**Table 2:** Clinical course of patients with suspected CSF leakage, all 18 patients.

Patient No	Sex	Age	Disease	Side	Offending Vessel	Mastoid Air Cell Exposure	Symptom from POD	LD from POD	LD Maintained	Hospital Stay	Symptom Recurrence
2021-95	F	36	HFS	R	PICA	+	2	-	-	9	-
2021-97	F	39	HFS	R	AICA	+	2	3	5	13	-
2021-106	M	69	HFS	L	AICA-PICA	+	3	-	-	7	-
2022-6	M	63	HFS	L	AICA-PICA-VA	+	5	6	6	15	-
2022-22	F	30	HFS	L	AICA-PICA	+	2	3	6	13	-
2022-59	M	63	HFS	L	PICA	+	3	-	-	9	-
2022-78	M	38	HFS	R	AICA	+	6	8	5	19	-
2022-91	F	77	TN	R	Vein	+	3	-	-	13	-
2022-99	M	39	HFS	L	AICA	+	3	-	-	8	-
2022-107	F	55	HFS	R	AICA	+	4	-	-	7	-
2022-117	F	56	HFS	L	AICA	+	4	-	-	8	-
2022-140	F	59	HFS	R	AICA-VA	+	5	6	4	14	-
2022-144	M	47	HFS	L	AICA-PICA-VA	+	2	-	-	8	-
2022-145	F	59	HFS	R	AICA	+	3	-	-	8	-
2023-4	M	53	HFS	L	AICA-PICA	+	3	-	-	7	-
2023-19	F	53	HFS	R	AICA	+	3	-	-	7	-
2023-30	F	69	HFS	L	VA	+	3	-	-	8	-
2023-33	F	55	HFS	L	AICA	+	3	-	-	8	-

CSF, cerebrospinal fluid; POD, postoperative day; M, male; F, female; R, right; L, left; HFS, hemifacial spasm; TN, trigeminal neuralgia; LD, lumbar drain.

**Table 3:** Patient characteristics with suspected CSF leakage.

**Suspected CSF leakage (N = 18)**	
Median age, years (range)	55 (30–77)
Male:Female	7:11
Left:Right	10:8
Symptom from POD	3.3 (2–6)
Mastoid air cell opened	18 (100%)
**Definite CSF leakage (N = 5)**	
Male:Female	2:3
Left:Right	2:3
LD catheter insertion	5 (100%)
LD insertion from POD	5.2 (3–8)
Maintenance period of LD catheter	5.2 (4–6)
Average hospital stay	14.8 (13–19)
CSF leakage symptom recurrence	0 (0.0%)
Revision operation	0 (0.0%)
Intracranial infection	0 (0.0%)

CSF, cerebrospinal fluid; LD, lumbar drain; POD, postoperative day.

## Data Availability

All data included in this study can be provided by contacting hs5937@hanmail.net.
